# Supplementary material for: Transcriptomic Analysis of Tobacco Plants in Response to Whitefly Infection
Source: Genes (Basel). 2023 Aug 18;14(8):1640. doi: 10.3390/genes14081640 (PMC10454835; doi:10.3390/genes14081640)
Supplement: Supplementary file 1 [file genes-14-01640-s001.zip › genes-2534179-supplementary.pdf]

Supplementary Figure

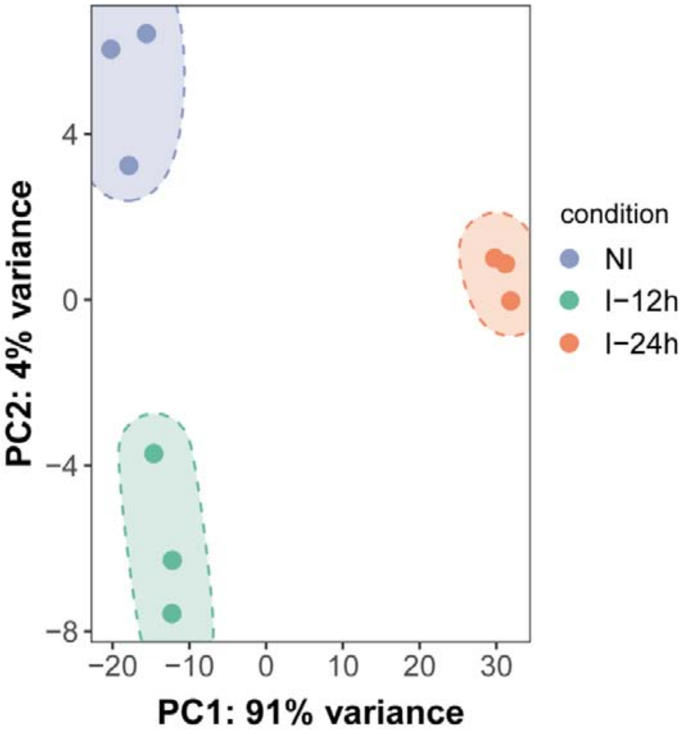

Fig. S1 Principal component analysis (PCA) of gene expression patterns in tobacco plants with different treatments. The first two principal components (PC1 and PC2) based on transcriptomic results are shown.

Table S1. Ten genes utilized for qRT-PCR validation

Gene name	Annotation
0000318g0020.1	Zinc finger CCCH domain-containing protein
0000722g0100.1	Ribulose-1,5-bisphosphate carboxylase_oxygenase activase 1
0000306g0200.1	ABC transporter G family member 40
0000202g0460.1	MYB transcription factor REVEILLE 8
0004821g0020.1	MYB transcription factor REVEILLE 1
0000116g0500.1	Late embryogenesis abundant protein 5
0000786g0090.1	L-ascorbate peroxidase
0003380g0010.1	Cryptochrome 1b
0002820g0100.1	Late embryogenesis abundant protein 5
0004814g0010.1	protein kinase At1g79600

Table S2. Primers used in this study

Genes	Forward primer (5′-3′)	Reverse primer (5′-3′)
**Primers used in qRT-PCR**		
Nttubulin	AAGTACATGGCTTGCTGCCT	ATCAATGCGCGAGAAGACCT
0000318g0020.1	GTGGATCGTTGAGTCTGAA	GGTAGTTGGAAGGCTCATAA
0000722g0100.1	CAATACACTGTCAACAACCA	TTCTCCATACGACCATCAC
0000306g0200.1	CAAGGTGCTGGTGAAGATA	GATGTCGCCGTCAATGTA
0000202g0460.1	CTTGAAGGTCCAGAAGAATG	AGAGTACAGAAGTGTCATCC
0004821g0020.1	GACAGAAGAAGAGCACAGAA	GCATCACCACTATTAGATTCAC
0000116g0500.1	CTCCACAATCAATTCCTTCC	TATCCACGCCTGCTAAGA
0000786g0090.1	GCTTGCTGCTGCTATGAA	CTTCTCTACATTACCACCACAT
0003380g0010.1	GAACTGAAGATGAAGAATCCTC	TCCAATCCTCCTACTTACCA

0002820g0100.1	CAGGTGGAGTGAGAGGAT	TTGTGGTTCAAGAGCATCT
0004814g0010.1	CGATTGGAGCAAGGTGAT	GAAGGCATACGGATGGAAT
